# Myeloid HDAC3 deletion protects against traumatic optic injury

**DOI:** 10.1038/s41420-026-03030-0

**Published:** 2026-03-18

**Authors:** Rami A. Shahror, Carol A. Morris, Ashlynn Cunningham, Piyanan Chuesiang, Abdelrahman Y. Fouda

**Affiliations:** 1 Department of Pharmacology & Toxicology, College of Medicine, University of Arkansas for Medical Sciences, Little Rock, AR USA; 2 Department of Clinical Pharmacy, Faculty of Pharmacy, Cairo University, Cairo, Egypt

**Keywords:** Regeneration and repair in the nervous system, Microglia

## Abstract

Traumatic optic neuropathy (TON) occurs due to trauma to the optic nerve, resulting in blindness. Current management focuses primarily on supportive care, highlighting an urgent need to identify novel treatment targets. Neuronal expression of the enzyme histone deacetylase 3 (HDAC3) has been previously implicated in retinal ganglion cell (RGC) degeneration after optic nerve crush (ONC), a model of TON. Here we investigated the role of myeloid HDAC3 (i.e., HDAC3 expressed in microglia and macrophages) in RGC loss, axonal degeneration, and efferocytosis, a reparative process by which phagocytic myeloid cells engulf apoptotic cells. ONC injury was performed on myeloid-specific HDAC3 knockout (KO) and floxed control mice. Neurodegeneration and efferocytosis assays were assessed using retina flatmount immunolabeling and confocal imaging. RGC function was evaluated using pattern electroretinography (PERG). Axonal sprouting was quantified by anterograde transport of cholera toxin B injected intravitreally. Myelin debris clearance was assessed in optic nerves in vivo and in vitro using bone-marrow-derived macrophages isolated from myeloid HDAC3 KO and control mice. Myeloid HDAC3 deletion preserved RGC and improved axonal regeneration after ONC, together with improved retinal function assessed by PERG. Furthermore, the deletion of HDAC3 enhanced the phagocytic function of myeloid cells to effectively remove apoptotic cells and myelin debris, both in vivo and in vitro. These protective effects were associated with the deletion of HDAC3 specifically in macrophages, since microglial-only deletion of HDAC3 did not preserve RGC count or function. The enhanced efferocytosis function of HDAC3 KO macrophages was at least partly dependent on increasing the expression of the phagocytic tyrosine kinase receptor, MerTK. The deletion of myeloid HDAC3 enhances efferocytosis, leading to neuroprotection, regeneration, and functional recovery following ONC. Targeting myeloid-HDAC3 presents a novel therapeutic strategy for TON.

## Introduction

Traumatic optic neuropathy (TON) is a type of optic injury that can potentially lead to vision loss. Direct or indirect trauma to the optic nerve triggers a progressive neurodegenerative process. Retrograde axonal degeneration results in retinal ganglion cells (RGC) loss over several days to weeks [1]. Following trauma, primary injury occurs due to immediate RGC axon damage that leads to cell death. Secondary injury, including optic nerve swelling and neuroinflammation, evolves after the primary injury and contributes to further apoptotic cell death of the surviving RGC [2–4].

To date, there is no effective treatment to stop axonal degeneration and RGC loss after TON, partly due to the incomplete understanding of the detailed underlying molecular mechanisms. The mouse model of optic nerve crush (ONC) injury is the most commonly used animal model to investigate the pathological processes that follow TON [5]. Studies have shown a critical role of microglia and resident/infiltrating macrophages in the pathophysiology of ONC [6–8]. Microglia and macrophages, collectively referred to as myeloid cells, can exhibit both pro-inflammatory and anti-inflammatory functions. In addition to modulating inflammation, myeloid cells can phagocytose apoptotic cells, as well as cellular and myelin debris, after injury through a programmed reparative process termed efferocytosis [9–12]. Hence, investigating and understanding the molecular mechanisms of myeloid cell-mediated efferocytosis following TON is essential for developing treatments for this condition.

Histones are key cellular proteins that control gene expression by maintaining chromatin structure in the nucleosome of eukaryotic cells [13, 14]. Histone deacetylases (HDACs) are a class of enzymes that regulate gene expression by deacetylating histone proteins, affecting chromatin’s structure and ultimately regulating gene expression. HDACs have been implicated in ONC injury, and their inhibition was shown to be protective [15]. One of the HDACs studied in the context of TON is HDAC3. Neuronal HDAC3 has been shown to contribute to RGC degeneration after ONC, as RGC-specific deletion of HDAC3 ameliorates RGC death [15]. Similarly, pharmacological inhibition of HDAC3 using the selective inhibitor RGFP966 was shown to be neuroprotective [16]. However, despite being a key contributor to degeneration in RGC, it remains unclear whether HDAC3 is involved in myeloid cell response following ONC. We have recently shown that myeloid HDAC3 plays a deleterious role in retinal ischemia by suppressing reparative efferocytosis [17]. Whether HDAC3 plays a similar role after ONC remains unknown.

Mer tyrosine kinase receptor (MerTK) belongs to the TAM family (Tyro3, Axl, and MerTK), which are receptor tyrosine kinases activated by the ligands growth arrest-specific gene 6 (Gas6) and Protein S to regulate vital processes like inflammation resolution, immune cell function, and efferocytosis [18]. MerTK plays a pivotal role in the functions of microglia and macrophages, such as the phagocytosis of apoptotic cells and myelin debris, and regulates the expression of anti-inflammatory markers [19–22]. Furthermore, MerTK signaling regulates retinal pigment epithelial (RPE) phagocytic function under physiological conditions by coordinating the necessary cytoskeletal modifications required for RPE cells to engulf photoreceptor outer segments [23–25]. Therefore, mutations in the MerTK gene in humans have been linked to retinitis pigmentosa and a variety of other ocular disorders affecting the photoreceptors [26, 27]. However, the role of myeloid MerTK in ONC or its regulation via HDAC3 signaling following injury has not been investigated before.

In the current study, we used myeloid HDAC3 knockout (KO) mice to investigate the role of myeloid HDAC3 in ONC injury by combining histological analyses with functional assessments and efferocytosis assays in vivo and in vitro. We also investigated the role of myeloid HDAC3 in MerTK expression and MerTK-mediated myelin efferocytosis. Our results demonstrated a deleterious role of myeloid HDAC3 in the ONC model. Myeloid HDAC3 deletion preserved RGC number and function, and facilitated axonal regeneration after injury through enhancing efferocytosis and debris clearance via MerTK.

## Results

### Deletion of myeloid HDAC3 protects retinal neurons from ONC injury

**Fig. 1:**
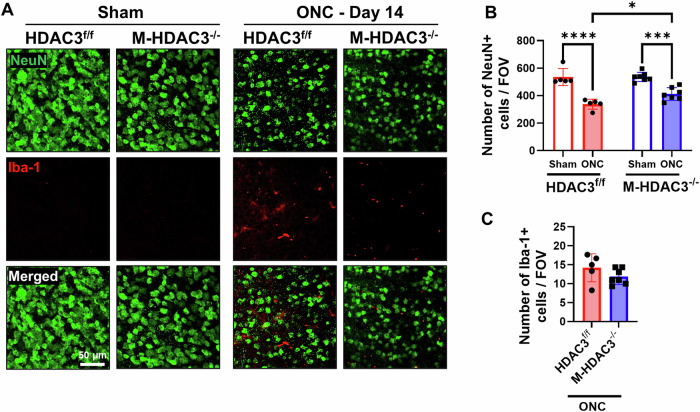
Myeloid HDAC3 deletion ameliorates ONC-induced neuronal cell loss. Representative images of retina flatmount immunolabeling (**A**) at 14 days post-injury and quantitative analyses (**B**) demonstrate decreased neurodegeneration indicated by the neuronal marker NeuN and a reduction in microglia/ macrophage numbers, marked by Iba-1 (**C**) in the M-HDAC3^−/−^ retinas (*N* = 5) compared to control HDAC3^f/f^ retinas (*N* = 7). FOV = Field of view, *p < 0.05, ***p < 0.005, ****p < 0.001.

The ONC model is widely used to simulate TON. We performed ONC on constitutive myeloid (microglia and macrophages) HDAC3 KO mice (M-HDAC3^−/−^) and floxed littermate controls (HDAC3^f/f^) to induce neurodegeneration and microglial activation in the retina and optic nerve [1]. We then examined neurodegeneration and microglial response at day 14 after ONC injury in retinal flatmounts by immunolabeling for the neuronal marker, NeuN, and the microglia/macrophage marker, Iba-1, using our established protocol, followed by confocal microscopy [17]. ONC led to neuronal degeneration, as indicated by a reduction in the NeuN count in the retinas of floxed mice (*p* < 0.001). Retinas from M-HDAC3^−/−^ mice showed reduced neurodegeneration as compared to HDAC3^f/f^ mice retinas (*p* < 0.05) (Fig. 1A, B). Microglia and macrophages exhibited a marked increase in count following ONC, as evidenced by Iba-1 labeling. The M-HDAC3^−/−^ retinas showed a trend toward a decrease in Iba-1 count after crush; however, this did not reach statistical significance (Fig. 1C). OCT conducted on day 7 and day 14 post-ONC revealed no difference in the thickness of the retinal ganglion cell complex (GCC) in the injured M-HDAC3^−/−^ mice as compared to the injured HDAC3^f/f^ control group (Supplementary Fig. S1A–D).

### Deletion of myeloid HDAC3 preserves retinal function after ONC injury

**Fig. 2:**
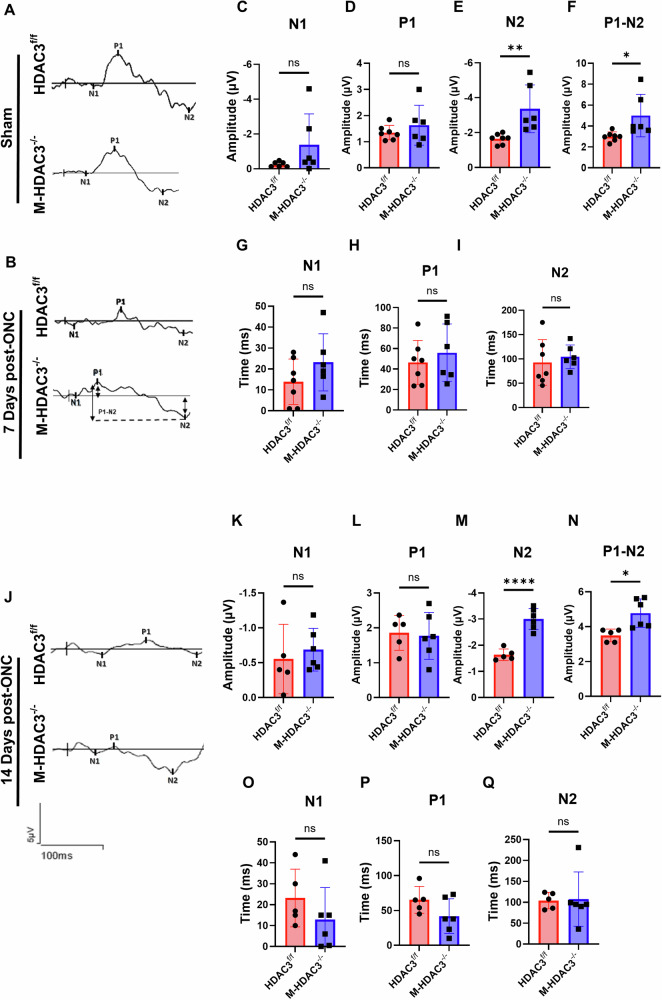
The deletion of myeloid HDAC3 improves PERG following ONC. **A**, **B** Representative N1, P1, and N2 waveforms in the retinas of HDAC3^f/f^ and M-HDAC3^−/−^ sham and injured mice, conducted on day 7 post-ONC injury. Quantification and comparison of the ONC groups reveal improved waveform amplitudes in M-HDAC3^−/−^ retinas with statistical significance achieved in N2 and P1-N2 amplitudes compared to HDAC3^f/f^ retinas at 7 days post-ONC injury (HDAC3^f/f^, *N* *=* *6*; M-HDAC3^−/−^, *N* *=* *7*)(**C**–**F**), with no effect on the wave latencies (**G**–**I**). Similarly, N2 and P1-N2 amplitudes were significantly improved (**J**–**N**), but not their latencies (**O**–**Q**) at 14 days post-ONC injury (HDAC3^f/f^, *N* = 5; M-HDAC3^−/−^, *N* = 6). *p < 0.05, **p < 0.01, ****p < 0.001, ns not statistically significant.

To test whether the observed retinal neuroprotection in M-HDAC3^−/−^ mice translates to functional improvement, we used PERG to measure the retinal function of M-HDAC3^−/−^ and floxed control mice at 7 and 14 days post-ONC injury. The PERG signal is primarily dependent on the activity of RGC, which reflects both cellular function and the connectivity of the inner retina circuitry [28]. Measurements included analysis of a small negative wave (N1), a positive wave (P1), and a prominent, broad negative wave (N2). No significant differences were observed in PERG waves or latencies between the uninjured sham groups (Fig. 2A and Supplementary Fig. S2A–G). M-HDAC3^−/−^ and HDAC3^f/f^ mice showed no difference in PERG amplitudes of the N1 and P1 waves at 7 days post-injury (Fig. 2B–D). However, myeloid HDAC3 deletion resulted in substantially improved PERG N2 wave amplitude as well as the full PERG amplitude measured from the P1 positive peak to the negative trough of N2 (*p* < 0.01 and *p* < 0.05, respectively) (Fig. 2E, F), suggesting that myeloid HDAC3 deletion preserves the retinal neuronal function after ONC. PERG waves corresponding latencies were not different between HDAC3^f/f^ and M-HDAC3^−/−^ ONC-injured mice (Fig. 2G–I). Similar, N1 and P1 were not different (Fig. 2J–L), whereas M-HDAC3^−/−^ mice showed enhanced PERG N2 wave (*p* < 0.001) and P1-N2 amplitude (*p* < 0.05) (Fig. 2M, N) at 14 days post-ONC with no effect on latencies (Fig. 2O–Q). Collectively, these results suggest that myeloid HDAC3 deletion protects against ONC, leading to neuroprotection and preservation of RGC function.

### Myeloid HDAC3 deletion improves apoptotic cell clearance after ONC injury

**Fig. 3:**
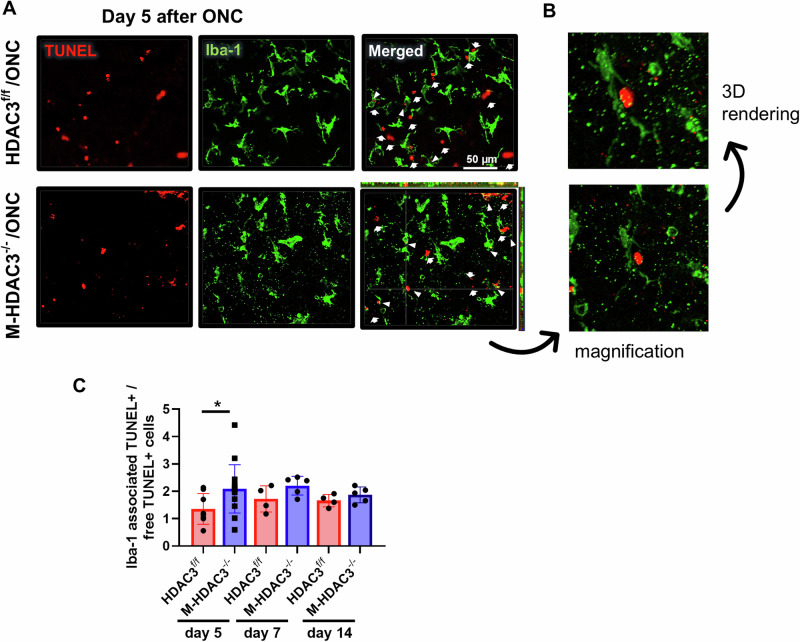
M-HDAC3 deletion enhances efferocytosis in vivo after ONC. M-HDAC3^−/−^ and HDAC3^f/f^ mice were subjected to ONC, and retinas were collected at days 5 (HDAC3^f/f^, *N* = 8; M-HDAC3^−/−^, *N* = 15), 7, and 14 days (HDAC3^f/f^, *N* = 4; M-HDAC3^−/−^, *N* = 5) post-injury. **A** Representative Z-Stack confocal images of retina flatmounts at day 5 post-ONC injury display colocalization of TUNEL^+^ apoptotic cells (red) and Iba-1^+^ microglia/macrophages (green). Arrows indicate free TUNEL^+^ apoptotic cells, while arrowheads denote Iba-1-associated apoptotic cells. **B** Magnification of the crosshair area from the orthogonal view and 3D rendering showing a myeloid cell wrapping its processes around an apoptotic cell. **C** The ratio of engulfed apoptotic cells by microglia/macrophages (Iba-1^+^ TUNEL^+^) to free apoptotic cells was markedly increased in injured retinas of M-HDAC3^−/−^ mice compared to HDAC3^f/f^ mice, indicating improved efferocytosis on day 5 after ONC. *p < 0.05.

Retinal ONC-induced neuronal injury results in progressive cell death by apoptosis [6]. Concurrently, myeloid cells are activated and proliferate [29–31]. We previously reported that HDAC3 deletion enhances myeloid cell-mediated efferocytosis after retinal ischemia-reperfusion injury [17]. Here, we sought to determine if HDAC3 deletion augments myeloid cell efferocytosis in the retina at 5, 7 and 14 days post-ONC. Immunofluorescent labeling of retina flatmounts after ONC showed co-localization of TUNEL-labeled apoptotic cells with Iba-1-labeled myeloid cells (Fig. 3A, B). M-HDAC3^−/−^ injured retinas showed enhanced efferocytosis, indicated by a significantly increase (*p* < 0.05) in Iba-1^+^ myeloid cell co-localization with TUNEL^+^ apoptotic cells at day 5 after injury, with a similar trend at days 7 and 14 that did not reach statistical significance (Fig. 3C). Collectively, these findings suggest that myeloid deletion of HDAC3 skews activated macrophages towards a phagocytic phenotype, enhancing the efferocytosis process after ONC injury, and that this effect is most pronounced at day 5.

### Myeloid HDAC3 deletion augments phagocytic macrophages and regeneration of RGC axons after ONC

**Fig. 4:**
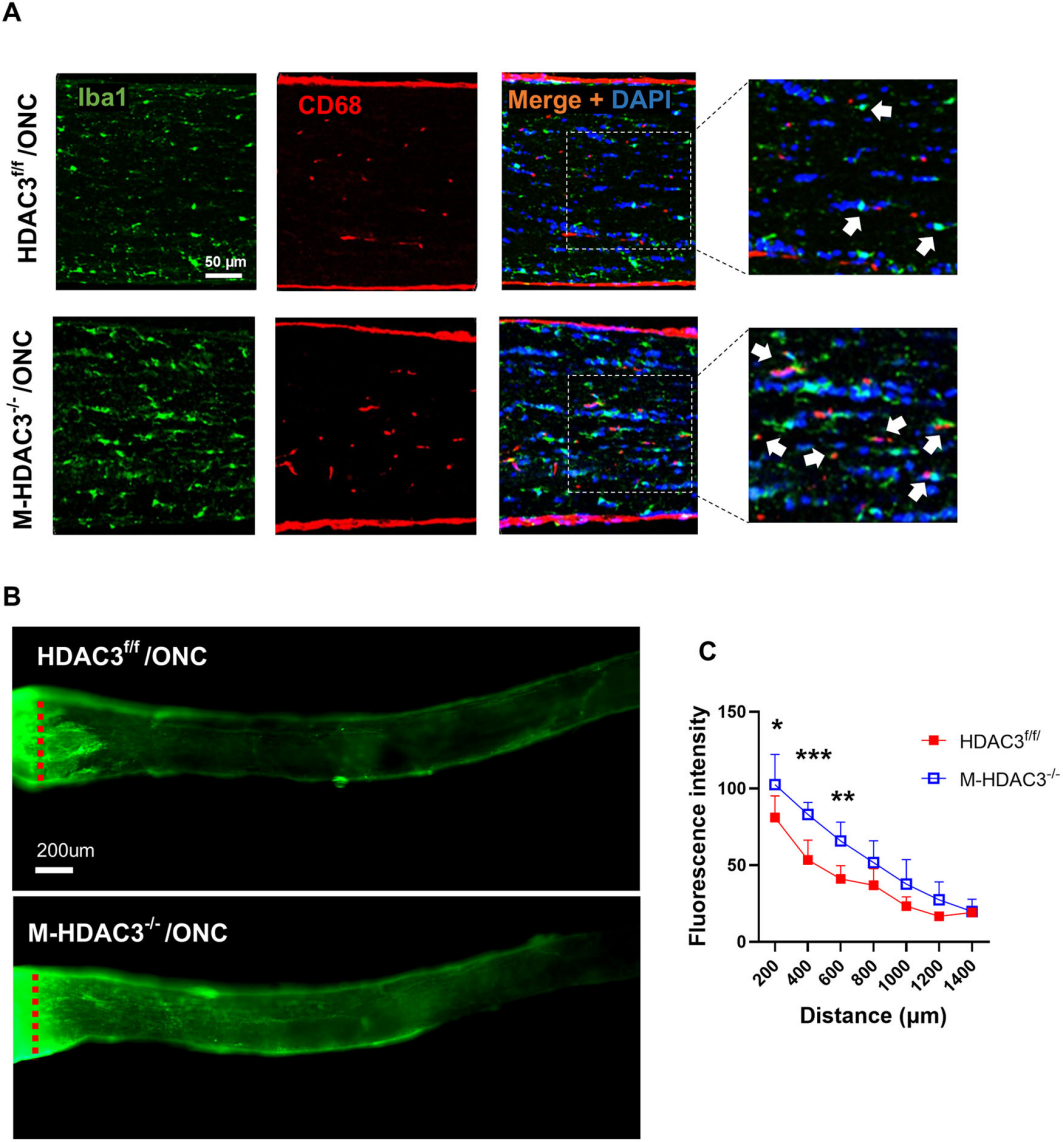
Deletion of myeloid HDAC3 increases phagocytic myeloid cells within the optic nerve and stimulates axonal regeneration after ONC. **A** Representative confocal images of optic nerve sections from M-HDAC3^−/−^ and HDAC3^f/f^ mice immunolabeled with Iba-1 (myeloid cell marker, green), CD68 (phagocytic cell marker, red), and DAPI (nuclei marker, blue) demonstrate an increase in phagocytic myeloid cells, indicated by arrows, in M-HDAC3^−/−^ mice compared to HDAC3^f/f^ on day 7 after ONC. **B** Representative confocal images of axonal growth and nerve fiber sprouting in the axons distal to the crush site by anterograde tracing with cholera toxin B (CTB) on day 14 post-ONC. **C** The quantification of the sprouting axons demonstrated significant improvement in axonal growth in M-HDAC3^−/−^ compared to HDAC3^f/f^ retinas, indicated by fluorescence intensity at distances of 200, 400, and 600 μm beyond the crush site (HDAC3^f/f^, *N* = 5; M-HDAC3^−/−^, *N* = 7). *p < 0.05, **p < 0.01, ***p < 0.005.

Next, we examined the impact of myeloid HDAC3 deletion on the prevalence of phagocytic myeloid cells in the crushed optic nerve. Immunolabeling of injured optic nerve sections with CD68 (a marker of phagocytic cells) and Iba-1 (to mark microglia/macrophages) revealed an increase in phagocytic myeloid cells (Iba-1^+^ /CD68^+^) in the injured M-HDAC3^−/−^ axons compared to the injured floxed controls (Fig. 4A) at day 7 post-ONC injury. We then labeled the regenerating axons by anterograde transport of cholera toxin B (CTB) to examine the axonal sprouting and regrowth beyond the crush site at day 14 post-injury. M-HDAC3^−/−^ mice showed a significant increase in regenerating axons at 200 (*p* < 0.05), 400 (*p* < 0.005), and 600 μm (*p* < 0.01) from the crush site, compared with the floxed control (Fig. 4B, C). These findings collectively suggest that myeloid HDAC3 deletion enhances axonal sprouting following ONC. This could be due to its influence on the phagocytic phenotype of myeloid cells in the optic nerve after crush, where myelin debris accumulates and hinders axonal regeneration.

### Myeloid HDAC3 deletion enhances myelin uptake in vitro

**Fig. 5:**
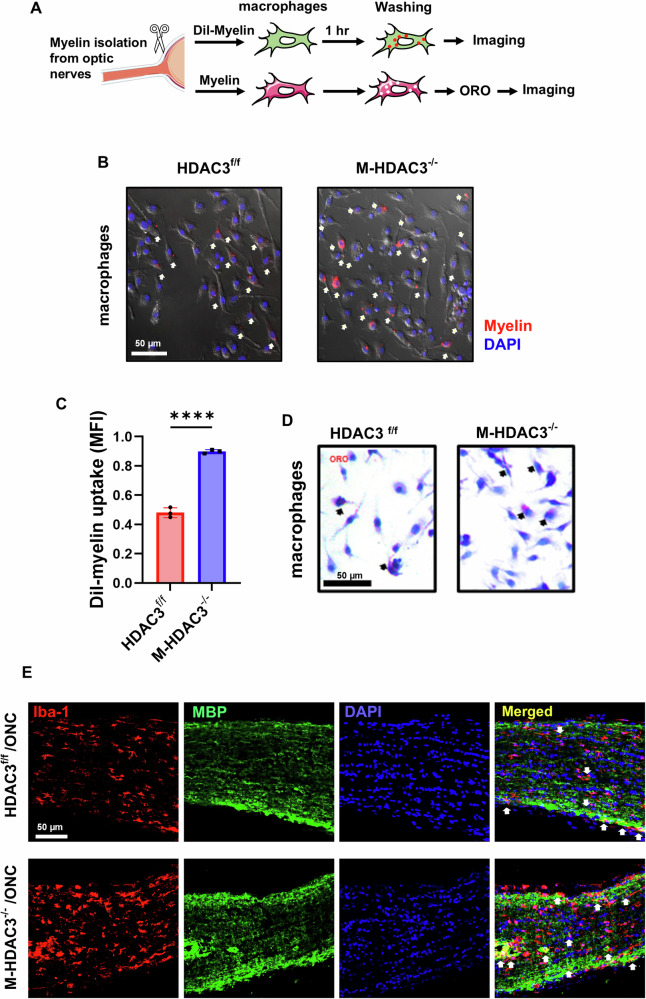
Myeloid HDAC3 deletion improves myelin internalization by macrophages in vitro and in vivo. **A** Experimental setup using myelin debris from the optic nerve labeled with Dil-red dye (top) and unlabeled debris stained with Oil Red O (ORO, bottom). **B** Representative images show the internalization of DiI-labeled myelin (arrows) by bone-marrow-derived macrophages derived from HDAC3^f/f^ and M-HDAC3^−/−^ mice. **C** Quantification of uptake of DiI-labeled myelin, expressed as mean fluorescence intensity (MFI), demonstrates significant improvement in the phagocytic activity of M-HDAC3^−/−^ macrophages compared to HDAC3^f/f^ macrophages. **D** ORO staining confirmed the improved uptake of myelin debris (arrows) by M-HDAC3^−/−^ macrophages compared to HDAC3^f/f^ macrophages. **E** Representative confocal images of Iba-1+ myeloid cells (red) and myelin basic protein (MBP, green) in optic nerve sections 7 days after ONC show a considerable increase in myelin clearance by myeloid cells in M-HDAC3^−/−^ optic nerves compared to HDAC3^f/f^ injured controls, as evidenced by increased Iba-1/MBP colocalization. *N* = 3 per group, ****p < 0.001.

Considering the substantial presence of myelin debris following ONC injury, and the detection of the phagocytic Iba-1^+^/CD68^+^ cells within the optic nerve (Fig. 4A), subsequent investigations were conducted to ascertain whether HDAC3 regulates the uptake of myelin by macrophages in vitro (see experimental design in Fig. 5A). Macrophages derived from M-HDAC3^−/−^ mice demonstrated enhanced uptake of Dil pre-labeled myelin, *p* < 0.001, compared to control cells (Fig. 5B, C). Oil Red O staining further validated the internalization of myelin by macrophages (Fig. 5D). In vivo, Myeloid HDAC3 deletion led to enhanced myelin debris clearance in M-HDAC3^−/−^ mice compared to flox controls, as shown by immunolabeling of optic nerve sections (Fig. 5E) for Iba-1 and myelin basic protein (MBP) at day 7 post-ONC. These results suggest that HDAC3 deletion enhances the clearance of myelin debris by myeloid cells.

### HDAC3 deletion upregulates MerTK in macrophages

**Fig. 6:**
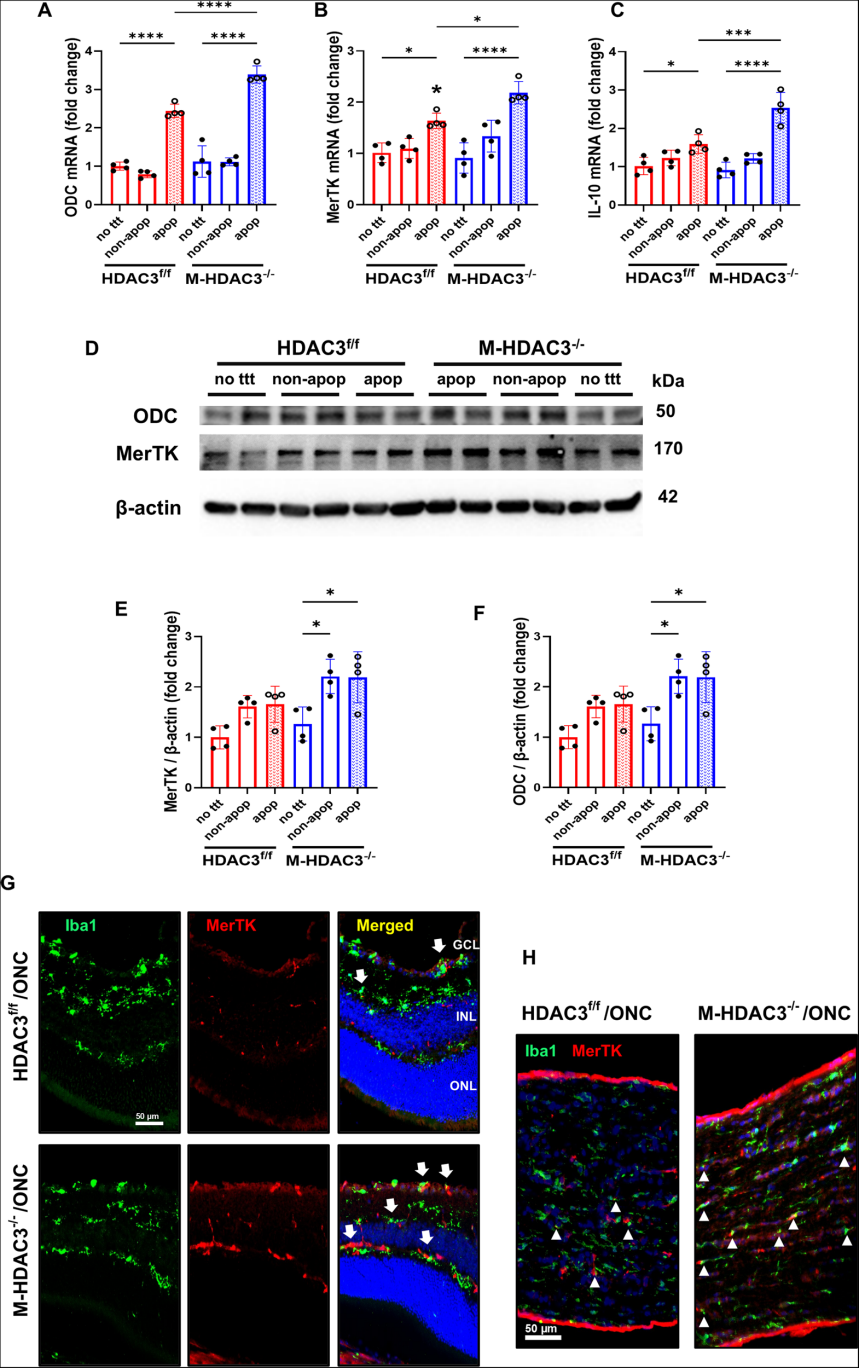
Deletion of myeloid HDAC3 upregulates MerTK expression. Macrophages from HDAC3^f/f^ and M-HDAC3^−/−^ mice were co-incubated with K-562 apoptotic cells (apop) in an in vitro efferocytosis assay. Controls included either co-incubation of macrophages with K-562 non-apoptotic cells (non-apop) or no treatment (no ttt). **A**–**C** Quantification of mRNA levels of ODC, MerTK, and the anti-inflammatory cytokine IL-10 demonstrated significant upregulation in M-HDAC3^−/−^ macrophages as compared to HDAC3^f/f^ macrophages incubated with apoptotic cells. **D**–**F** Western blotting shows significant upregulation of MerTK and ODC in M-HDAC3^−/−^ macrophages co-cultured with K-562 cells compared to untreated M-HDAC3^−/−^ macrophages, but not in the HDAC3^f/f^ control co-cultures. β-actin was used as a loading control. **G** Representative confocal images of Iba-1^+^ myeloid cells (green) and MerTK (red) in retinal sections 7 days after ONC show a considerable increase in MerTK expression by myeloid cells in M-HDAC3^−/−^ retinas compared to HDAC3^f/f^ controls, as evidenced by increased Iba-1/MerTK colocalization. **H** Similarly, MerTK expression by myeloid cells is increased in injured optic nerve sections, with arrowheads pointing to Iba-1^+^ MerTK^+^ myeloid cells. GCl ganglion cell layer, INL inner nuclear layer, ONL outer nuclear layer. *N* = 4 per group. *p < 0.05, ***p < 0.005, ****p < 0.001.

Our recent studies have shown upregulation of arginase 1 (A1) in HDAC3^−/−^ macrophages, along with enhanced efferocytosis [17]. Other studies reported the involvement of A1 and its downstream enzyme ornithine decarboxylase (ODC) in efferocytosis and inflammation resolution via upregulation of the receptor, MerTK, and the anti-inflammatory cytokine interleukin 10 (IL-10) [32]. We subjected bone-marrow-derived macrophages from control and M-HDAC3 KO mice to apoptotic, non-apoptotic cells or no treatment. We then quantified mRNA levels of ODC, MerTK, and IL-10, which showed significant upregulation in M-HDAC3^−/−^ macrophages as compared to HDAC3^f/f^ macrophages incubated with apoptotic cells (Fig. 6A–C). Western blotting further confirmed the upregulation of ODC and MerTK in M-HDAC3^−/−^ macrophages incubated with apoptotic or non-apoptotic cells (Fig. 6D–F). Myeloid HDAC3 deletion led to increased MerTK expression in M-HDAC3^−/−^ mice myeloid cells compared to flox controls, as shown by immunolabeling of retina (Fig. 6G) and optic nerve sections (Fig. 6H) for Iba-1 and MerTK at day 5 post-ONC. These findings demonstrate, for the first time, an inhibitory effect of HDAC3 on MerTK expression in macrophages. However, the deletion of Myeloid HDAC3 did not affect MerTK expression by glial cells in the retina or optic nerve at day 5 following ONC (Supplementary Fig. S3A, B).

### MerTK mediates myelin uptake by HDAC3 KO macrophages

**Fig. 7:**
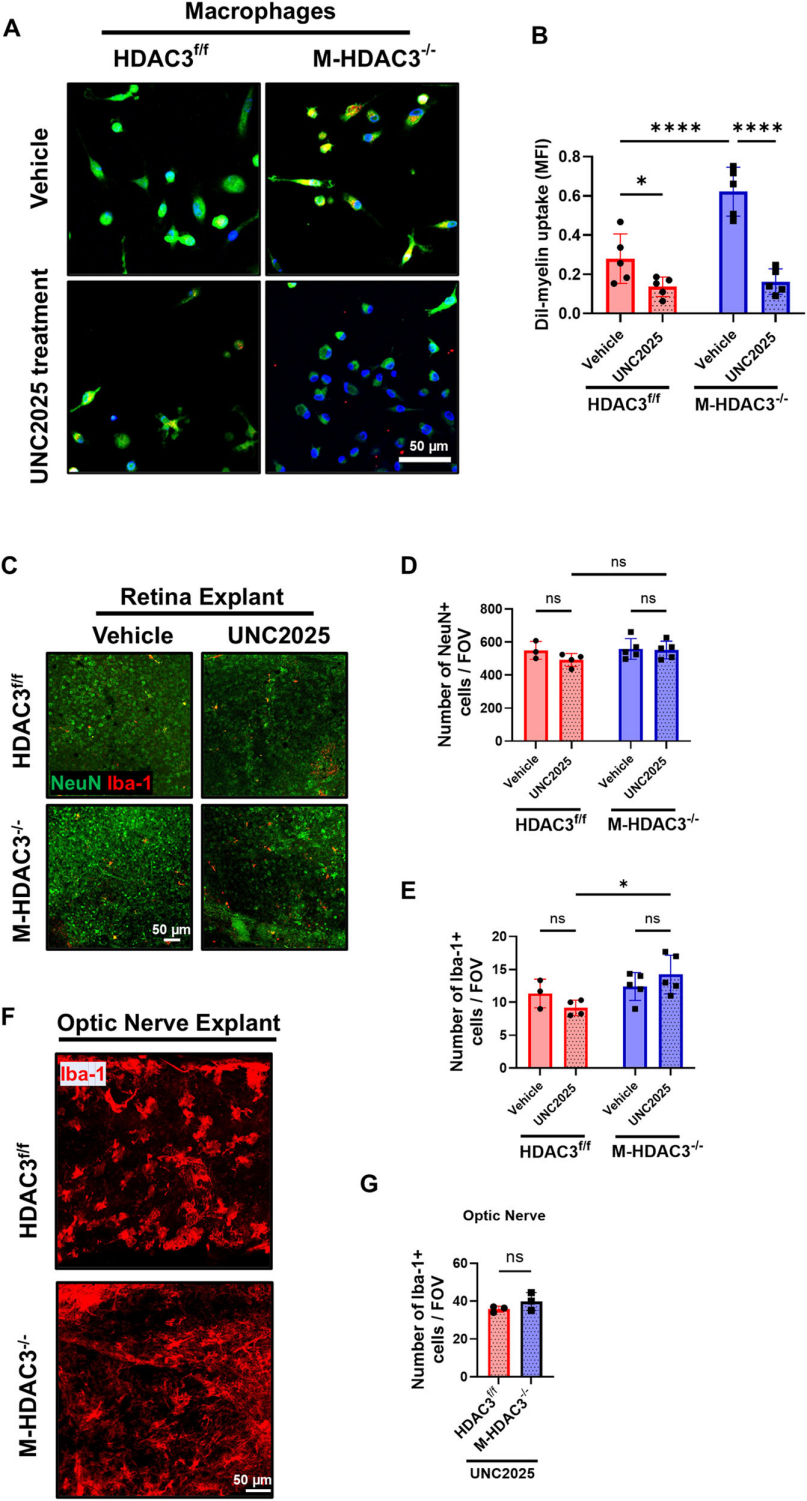
Myeloid HDAC3 deletion augments the clearance of myelin debris via MerTK. **A** Representative images illustrate the internalization of DiI-labeled myelin debris (red), derived from optic nerve axons, by CFDA-labeled macrophages (green) obtained from HDAC3^f/f^ and M-HDAC3^−/−^ mice (*N* = 5 per group). These cells were pretreated with 0.8 nM of the MertK inhibitor (UNC2025) or vehicle for 1 h in vitro. **B** Quantification of DiI-labeled myelin debris uptake, expressed as mean fluorescence intensity (MFI), demonstrates a significant reduction in myelin uptake in the UNC2025 pretreatment groups, with UNC2025 abolishing the enhanced myelin uptake observed in vehicle-treated M-HDAC3^−/−^ macrophages, indicating that myeloid HDAC3 deletion promotes myelin uptake at least in part via MerTK. **C** Representative images of immunolabeling for neurons, marked by NeuN (green), and microglia/macrophages, marked by Iba-1 (red), of adult M-HDAC3^−/−^ and HDAC3^f/f^ mice retinas that were explanted for 24 h and treated with UNC2025 or vehicle (HDAC3^f/f^, *N* = 3, 4; M-HDAC3^−/−^, *N* = 5) for another 24 h. **D**, **E** Quantification of NeuN and Iba-1 shows that UNC2025 treatments had no significant effect on neuronal preservation in retinal explants of both treated groups, while it significantly increased myeloid cell number in M-HDAC3^−/−^ compared to flox retina explants. **F**, **G** Representative images of Iba-1 (red) labeling and quantification of optic nerve explants treated with UNC2025 show no effect of the treatment on myeloid cell count between M-HDAC3^−/−^ and HDAC3^f/f^ derived optic nerves (*N* = 3 per group). *p < 0.05, ****p < 0.001; ns, not statistically significant; FOV, field of view.

We then investigated the role of MerTK in HDAC3^−/−^ macrophages-mediated myelin debris clearance in vitro and RGC preservation ex vivo. In vitro, MerTK inhibition using UNC2025 significantly abrogated the enhanced myelin uptake by HDAC3^−/−^ macrophages (*p* < 0.001; Fig. 7A, B). These findings suggest that the deletion of HDAC3 in myeloid cells enhances the capacity of macrophages to uptake and clear myelin debris via the upregulation of MerTK expression.

We next used ex vivo retina/optic nerve explants to assess the effect of MerTK inhibition on RGC viability after axotomy. Retinal explants derived from M-HDAC3^−/−^ or floxed control mice showed similar neuronal count after 24 h of incubation ex vivo. Treatment with UNC2025 had no significant effect on RGC preservation in retinal explants derived from M-HDAC3^−/−^ or floxed control mice (Fig. 7C, D). Interestingly, the treatment with UNC2025 resulted in a substantial increase in the number of myeloid cells (indicated by Iba-1) in M-HDAC3^−/−^ retinal explants relative to floxed control explants (*p* < 0.05; Fig. 7E). However, this effect was not observed in the optic nerves of these explants (Fig. 7F, G). The lack of observed differences in neurodegeneration between the groups in the explant may be attributed to the model’s focus solely on the microglial response while excluding macrophage infiltration. In subsequent experiments, we investigated the role of microglial HDAC3 in vivo.

### Microglial-specific HDAC3 deletion does not affect RGC count or function following ONC

**Fig. 8:**
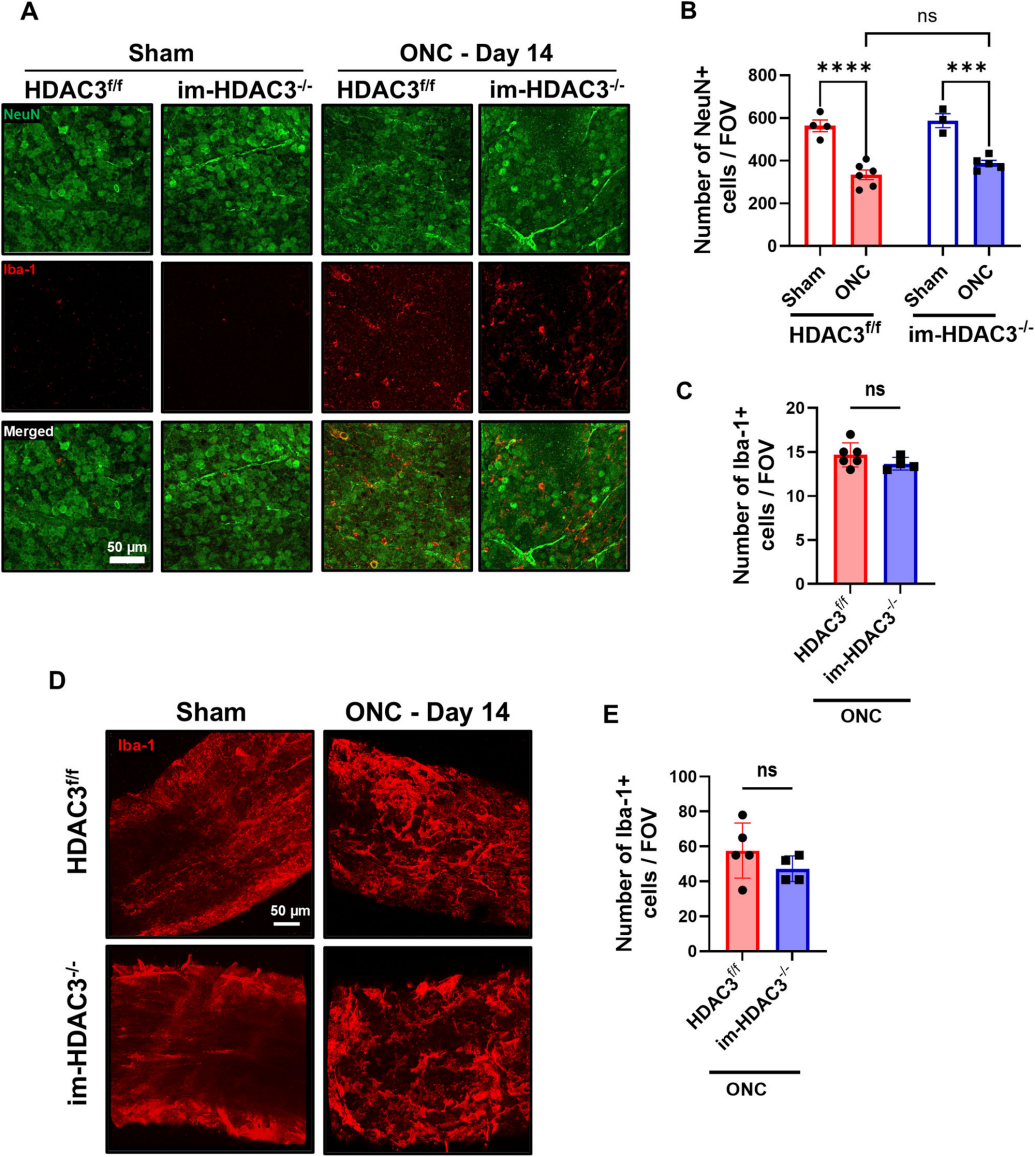
Microglial-specific deletion of HDAC3 does not affect ONC-induced neurodegeneration. **A** Representative retina flatmount images from microglia-specific HDAC3 KO (im-HDAC3^−/−^) and HDAC3^f/f^ controls immunolabeled for NeuN (neuronal marker, green) and Iba-1 (microglia/macrophages marker, red) at 14 days after ONC. **B**, **C** Quantitative analyses reveal significant neurodegeneration, indicated by a decrease in the neuronal marker NeuN and increase in Iba-1^+^ cell count in injured im-HDAC3^−/−^ (*N* = 5) and HDAC3^f/f^ (*N* = 6) mice compared to shams (*N* = 3 for im-HDAC3^−/−^, and *N* = 4 for HDAC3^f/f^). However, no differences were observed between the injured groups. **D**, **E** Representative images and Iba-1 quantification at the optic nerve injury site of im-HDAC3^−/−^ (*N* = 5) and HDAC3^f/f^ (*N* = 4) mice 14 days post-ONC injury show a robust presence of activated microglia and macrophages, with no differences observed between the injured groups. ***p < 0.005, ****p < 0.001; ns, not statistically significant; FOV, field of view.

ONC leads to microglial activation, which gradually increases over time, reaching a peak at day 14 after injury, and can be observed up to 2 months [33, 34]. This activation is associated with poor RGC survival [35]. We determined the role of microglial-specific HDAC3 deletion in RGC preservation at 14 days post-ONC using tamoxifen-inducible microglia-specific HDAC3 KO mice (im-HDAC3^−/−^). Tamoxifen was administered for 5 consecutive days, followed by a 30-day washout period to induce deletion only in microglia, which are long-lived, while allowing the replenishment of HDAC3 in rapidly renewing myeloid cells (monocytes/macrophages). We found a significant reduction in RGC (marked by NeuN) in the injured im-HDAC3^−/−^ and HDAC3^f/f^ mice as compared to shams. However, we found no differences between the injured groups (Fig. 8A, B). Furthermore, there was no significant difference in the number of microglia/macrophages (indicated by Iba-1) between the injured groups (Fig. 8C). Although the presence of activated microglia/macrophages was notably evident at the site of nerve crush (Fig. 8D), no differences were observed between the optic nerves of the injured groups (Fig. 8E). Next, we analyzed the effect of microglial-specific HDAC3 deletion on the RGC functions at 14 days post-ONC using PERG. No differences between the injured groups were observed in N1, P1, or N2 wave amplitudes (Supplementary Fig. S4A–E), or their latencies (Supplementary Fig. S4F–H). Based on the compelling results presented in Fig. 1, these findings suggest that the deletion of myeloid HDAC3 preserves RGC and their functions, and that this protective effect is mainly attributable to the macrophage subset of retinal myeloid cells, rather than microglia.

## Discussion

Our study demonstrates for the first time that myeloid HDAC3 contributes to neurodegeneration and hinders axonal regeneration following ONC. Additionally, the deletion of HDAC3 enhances the clearance of myelin debris and promotes efferocytosis by increasing the expression of the phagocytic receptor MerTK. These effects appear to be mediated by macrophages rather than microglia, suggesting that targeting this pathway can facilitate axonal regeneration and improve repair processes.

Immune response to traumatic central nervous system (CNS) injuries is dominated by myeloid cells that include microglia and macrophages [36]. In the CNS, activated macrophages can enhance regeneration by phagocytosing myelin debris [37–39]. After ONC, myeloid cells are recruited to the injury site [40]. Activated macrophages were shown to stimulate mature RGC to regenerate axons [41]. We have previously shown that stimulated macrophages lacking HDAC3 exhibit a decreased inflammatory response [42]. We have also shown that HDAC3 is upregulated in retinal myeloid cells after IR [42]. This upregulation impairs efferocytosis and worsens IR injury outcomes [17]. Based on our previous findings that myeloid HDAC3 inhibition or deletion enhances neuroprotection, functional recovery, and efferocytosis after IR, we sought to determine the role of myeloid HDAC3 in neurodegeneration and neuroregeneration, cellular and myelin efferocytosis, and visual functional recovery in a mouse model of ONC.

ONC injury induces progressive RGC degeneration [43]. A previous study has shown that RGC-specific HDAC3 mediates RGC death after ONC [15]. In the present study, we demonstrated that myeloid-specific HDAC3 deletion (M-HDAC3^−/−^) reduces RGC degeneration following ONC. We have used NeuN to assess neurodegeneration, as previous reports [44] and personal communications with other labs have indicated that RGC-specific markers, such as Rbpms and Brn3a, can be downregulated in viable RGC after crush. Since the model involves a crush to the optic nerve, the RGCs are the primary cells to be affected, and therefore, a decrease in NeuN signal correlates with RGC loss. Indeed, our previous work indicated that NeuN can be effectively used for ONC studies and follows the pattern of both Brn3a and Rbpms [6]. In addition to the retinal neuronal preservation, M-HDAC3^−/−^ injured mice showed enhanced axonal sprouting, evidenced by anterograde transport of CBT at 14 days after ONC. The preserved retinal neurons, along with axonal regeneration, resulted in improved retinal function in the M-HDAC3^−/−^ injured mice. The P1 and N2 components of the PERG recording after ONC are extensively dependent on the connectivity of the inner retina circuitry and RGC count [28, 45–47]. Our results showed an improved PERG amplitude of the N2 wave and P1-N2 in M-HDAC3^−/−^ mice on days 7 and 14 after ONC injury, which correlated with the preservation of RGC in retina flatmounts. These observations were associated with the deletion of HDAC3 specifically in macrophages, as demonstrated by the fact that microglial-only deletion of HDAC3 in im-HDAC3^−/−^ mice did not preserve RGC or improve their function.

The damaged optic nerves have abundant myelin debris after ONC. Myeloid cells facilitate the clearance of the inhibitory myelin debris and thus facilitate axonal regrowth [48, 49]. The number of Iba-1^+^ myeloid cells in the retina flatmount did not differ between groups, suggesting that HDAC3 deletion has no effect on myeloid cell proliferation or infiltration in the retina after ONC. Rather, we found enhanced efferocytosis activity of myeloid cells in M-HDAC3^−/−^ mice, evidenced by increased co-localization of Iba-1^+^ myeloid cells with TUNEL^+^ apoptotic cells in retinal flatmount at day 5 after ONC. Our data also indicate that deletion of HDAC3 in macrophages improved the clearance of myelin debris in vitro, together with increased phagocytic myeloid cells in vivo. Collectively, these results suggest that myeloid HDAC3 deletion directs the macrophages to a more reparative, pro-efferocytic phenotype.

We previously demonstrated that A1 upregulation in M-HDAC3^−/−^ macrophages was linked to enhanced efferocytosis [17]. Additionally, others showed that A1 and its downstream enzyme, ODC, play roles in efferocytosis and inflammation resolution by upregulating MerTK and the anti-inflammatory cytokine IL-10 [32]. In the present study, we demonstrated that M-HDAC3^−/−^ macrophages, when challenged with apoptotic cells, upregulate IL-10, MerTK, and ODC mRNA and we confirmed the elevation of the latter two at the protein levels. Additionally, we showed that myeloid HDAC3 deletion enhanced MerTK expression in myeloid cells of the retina and optic nerve after ONC. Additional in vitro experiments using MerTK inhibition indicated that the enhanced myelin debris clearance by HDAC3 KO macrophages is at least partly dependent on MerTK.

We evaluated the role of myeloid HDAC3 deletion and MerTK upregulation in retinal neurodegeneration using ex vivo experiments with retina explants as an axotomy model. We found that neither HDAC3 deletion nor MerTK inhibition affected neurodegeneration in the explants. The discrepancy between our findings in explants and those observed in vivo may be attributed to differences in injury, specifically, the crush injury used in vivo versus axotomy in ex vivo conditions. Additionally, the ex vivo explant preparation excludes the contribution of infiltrating macrophages, which could also account for the differences observed. Indeed, microglia-specific deletion of HDAC3 did not improve neurodegeneration after ONC.

While our findings provide important insights, certain limitations remain. The underlying mechanism by which HDAC3 regulates MerTK expression remains undefined and will be explored in future studies. Furthermore, we observed a phenotypic divergence between the constitutive myeloid KO mouse (M-HDAC3^−/−^) and the inducible, microglia-only KO mouse (im-HDAC3^−/−^), with the latter showing no protection against ONC. This discrepancy is particularly striking given that the im-HDAC3^−/−^ mouse yielded protective effects in a model of traumatic brain injury, thus suggesting highly context-dependent roles for HDAC3 across different neurological pathologies [50]. Future studies will clarify these differences and delineate the cell-specific roles of HDAC3 in microglial versus macrophage-driven efferocytosis.

## Conclusion

In conclusion, our findings demonstrate that myeloid HDAC3 deletion preserves RGC survival and function while promoting axonal regeneration. Mechanistically, loss of HDAC3 enhances efferocytosis in vivo and accelerates the clearance of apoptotic cells and myelin debris in vitro via MerTK upregulation. While the precise signaling cascades underlying these reparative effects post-ONC warrant further investigation, this study identifies myeloid HDAC3 as a critical therapeutic target. Inhibiting HDAC3 can drive macrophages toward a pro-reparative phenotype, providing a promising strategy for protecting and regenerating the injured optic nerve.

## Materials and methods

### Experimental animals

Animal experiments were approved by the UAMS Institutional Animal Care and Use Committee (IACUC). They were performed following the Association for Research in Vision and Ophthalmology (ARVO) Statement for the Use of Animals in Research. All mice were housed under a 12-h dark/light cycle, with access to food and water provided ad libitum. C57BL/6J floxed mice with LoxP sites flanking both sides of exons 7 of HDAC3 (HDAC3^f/f^), initially developed by Dr. Scott W. Hiebert and obtained from Dr. McGee-Lawrence, were bred in our facility [51]. Two myeloid HDAC3 KO lines were used (genotyping is included in Supplementary Fig. S5):

1. A constitutive macrophage and microglia cell-specific HDAC3 KO (LysM^Cre/+^; HDAC3^f/f^ or M-HDAC3^−/−^) mice generated by crossing the HDAC3^f/f^ mice with LysM^Cre/+^ mice (Jackson laboratory Stock # 004781). We have previously characterized these mice, which lack HDAC3 in myeloid cells (macrophages and 30% of microglia) and exhibit a normal retina phenotype [17].
2. An inducible microglia-only HDAC3 KO (Cx3cr1^CreER/+^; HDAC3^f/f^ or im-HDAC3^−/−^) colony generated by crossing HDAC3^f/f^ mice with tamoxifen-inducible Cx3cr1^CreER/+^ mice (Jackson laboratory stock # 020940). To accomplish the deletion of HDAC3 only in microglia, the im-HDAC3^−/−^ mice were treated with tamoxifen via intraperitoneal injection at a dosage of 75 mg/kg body weight daily for five consecutive days. This was followed by a 30-day washout period to permit the replenishment of HDAC3 in the rapidly renewing myeloid cells (monocyte/macrophages), whereas HDAC3 remained knocked out of microglia, which are long-lived. HDAC3^f/f^ mice, used as controls for the im-HDAC3^−/−^ experiments, were subjected to the same tamoxifen treatment protocol to account for any off-target effects due to tamoxifen administration. We have confirmed the HDAC3 deletion in microglia of these mice in Supplementary Fig. S6.

Experiments were conducted on male mice, aged 10–16 weeks at the time of the ONC induction. We randomized animals to experimental groups by an independent technician.

### ONC induction

For ONC induction, mice were anesthetized, and the standard ONC procedure was conducted as previously detailed [7]. All surgical tools were sterilized by autoclaving before surgery. Mice were anesthetized with isoflurane, and a single drop of topical anesthetic (0.5% proparacaine hydrochloride) was applied to the eye. Using forceps, the conjunctiva of the left eye was pinched, and a small incision was made on the temporal side. The orbital muscles were gently retracted to expose the optic nerve, ensuring the orbital sinus remained undamaged. The optic nerve was then clamped for 3 s at 1–2 mm from the eyeball using a self-closing N7 forceps (Fine Science Tools). The right eye was used as a sham control.

### Immunolabeling

Retinal samples were obtained as previously described by us [7]. Briefly, anesthetized mice with ketamine/xylazine were intracardially perfused with 4% paraformaldehyde (PFA) in 0.1% phosphate-buffered saline (PBS), pH 7.4. Eyeballs were removed and fixed in 4% PFA in 0.1 M phosphate buffer, pH 7.4, overnight at 4 °C. Eyeballs were processed for either retinal flatmounts or frozen sections.

For flatmounts, retinas were dissected out of the eyeballs. Retinas were then blocked and permeabilized using a blocking/permeabilization buffer that included 0.1% Triton X-100, 10% normal donkey serum, and 1% bovine serum albumin in PBS for 1 h. Primary antibodies used were Iba-1 (FUJIFILM Wako, Cat. #019-19741) and NeuN (MilliporeSigma, Cat. #BN78MI). Retinas were incubated overnight at 4 °C in primary antibodies diluted in the blocking buffer. After rinsing with PBS, retinas were incubated with secondary antibodies conjugated to either Alexa 488 or Alexa 594 at room temperature for 1 h, followed by PBS washing as described earlier by us [17].

For frozen sections, the eyeballs and optic nerves were cryoprotected in a 30% sucrose solution overnight, embedded in Optimal Cutting Temperature (O.C.T.) compound, and sectioned at a thickness of 10 μm using a cryostat. Sections were then labeled for Iba-1, CD68 (BioLegend, Cat. #137002), MerTk (R&D Systems, Cat. # AF591), MBP (Abcam, Cat. # ab7349), HDAC3 (Proteintech, Cat. # 10255-1-AP), and GFAP (Abcam, Cat. #13-0300). Retinal flatmounts and sections were then mounted and covered with Vectashield mounting medium (Vector Laboratories).

### Microscopy and quantification

Confocal micrographs were captured using an LSM880 Airyscan Zeiss Laser inverted microscope equipped with 405 nm, 488 nm, 561 nm, and 640 nm laser lines. The same laser intensity and acquisition settings were applied to all samples, and Z-stack images (resolution: 1024 × 1024 pixels) were taken. After the imaging, a maximum-intensity projection of the Z-stack was applied using ImageJ. Quantitative analysis was performed using ImageJ on Z-stacked confocal images, where cell count per field of view (FOV) was determined and compared.

### Optical coherence tomography (OCT)

Mice’s eyes were dilated with 1% tropicamide (Akron Pharmaceuticals), and their retinas were scanned using an OCT Ophthalmic Imaging System (Bioptigen Inc.) under ketamine/xylazine anesthesia. During scanning, mice were secured on a dedicated holder to maintain proper posture. The retina scans were taken in a rectangular volume mode, consisting of 3 frames per scan, with 1000 A-scans per B scan and 100 B scans (covering an area of 1.4 mm × 1.4 mm). Automated analysis with InVivoVue software (Bioptigen Inc., Durham, NC) was used to measure retinal thicknesses.

### In vivo efferocytosis assay

A microscopy-based in vivo efferocytosis assay was performed as we previously described [17]. Briefly, retinal flatmounts collected at day 5 after ONC were immunolabeled for the microglia/macrophage marker Iba-1, along with apoptotic cell labeling using the Click-iT™ Plus TUNEL assay kit (Invitrogen™, Cat. # C10618) according to the manufacturer’s instructions. Confocal microscopy-based quantification of the number of Iba-1^+^ cells associated with TUNEL^+^ apoptotic bodies was performed in multiple fields of view in Z-stack images taken at the ganglion cell layer (GCL). The clearance of dead/dying cells in the retinal flatmount was calculated as the efferocytosis index, which represents the ratio of dead cells engulfed by myeloid cells to total dead cells. The efferocytosis index was calculated using the following equation: (the mean number of Iba-1^+^ TUNEL^+^ cells ÷ the number of free TUNEL^+^ cells).

### Axonal regeneration and nerve fiber sprouting

Sprouting RGC axons in injured optic nerves distal to the crush site were identified by anterograde labeling with Alexa Fluor® 647-conjugated cholera toxin B (CTB) and quantified as we described previously with some modification [6, 7]. CTB was injected intravitreally into the injured eye (0.2%, 1 μg/eye) of the anesthetized animals at 14 days post-ONC. After 24 h, the CTB-treated animals were anesthetized and perfused, and the optic nerves were dissected. After fixation, the optic nerves were cleared using FocusClearTM, following the manufacturer’s recommendations. Regenerating axons visualized by the CTB tracer were quantified at distances of 0.2, 0.4, 0.6, 0.8, 1, 1.2, and 1.4 mm from the injury site.

### Electroretinography study

A pattern electroretinogram (PERG) was recorded at day 14 post-injury using the Celeris electroretinogram system (Diagnosys LLC). Briefly, dark-adapted mice were anesthetized with ketamine/xylazine, and pupils were dilated with 2.5% phenylephrine eye drops. Mice were then placed on the Celeris electroretinogram system platform with their body core temperature maintained at 37 °C using the built-in warming platform of the recording setup. A stimulus/recording electrode was placed directly in contact with the cornea. A reference electrode was placed on the other eye. The specialized recording electrode provides a reversing checkerboard pattern. Based on a standard machine protocol, the PERG amplitude is recorded as the difference between the positive peak (P1) and the negative peak (N2) in microvolts (μV).

### Myelin isolation and phagocytosis assay

Myelin was purified from mouse pooled optic nerve tissue (*N* = 6) using sucrose density gradient centrifugation, as described previously [52]. The myelin protein concentration was determined using the bicinchoninic acid (BCA) protein assay kit (Thermo Fisher, Cat. # C10618) according to the manufacturer’s guidelines. Bone-marrow-derived macrophages were isolated and differentiated as we previously described [17]. Then, macrophages were treated with 0.8 nM of the MerTK Inhibitor UNC2025 (Cayman Chemicals, Cat. # 50-296-5010) [53] or vehicle for 1 h, then treated with 30 μg/ml myelin in media for 1 h. To evaluate the extent of myelin phagocytosis, myelin was fluorescently labeled with Dil-red, and a CFDA green fluorescent dye (Thermo Fisher) was used to label macrophages. Images were acquired using confocal microscopy. The uptake of myelin was further confirmed by Oil Red O (ORO) staining, which stains neutral lipids. Briefly, macrophages were washed with PBS, then stained with 0.3% ORO for 10 min, followed by counterstaining with hematoxylin.

Another set of experiments involved incubating macrophages with K562 cells (either apoptotic or non-apoptotic) for 45 min at 37 °C to stimulate efferocytosis in vitro, followed by PBS washing to remove unengulfed K562 cells as we previously described [17]. Subsequently, the cells were incubated for an additional 6 or 18 h and then collected for mRNA and protein analysis, respectively. Apoptosis was induced in K562 cells using UV-B irradiation for 15 min in a UV crosslinker (Spectrolinker XL-1500, Spectronics Corporation, Melville, NY), as previously described [17].

### Ex vivo explants

Retinal explants, along with their optic nerves, were isolated to model axotomy following published protocols with modifications [7, 54, 55]. Mice were subjected to deep anesthesia using a ketamine/xylazine mixture and subsequently euthanized by cervical dislocation. Retinal eyecups were immersed in ice-cold Hanks’ balanced salt solution (HBSS) supplemented with penicillin (100 U/mL) and streptomycin (100 μg/mL) and meticulously dissected with their optic nerves intact under a dissecting microscope. Subsequently, the retinas were positioned within cell culture inserts (12 mm diameter, 0.4 μm pore size, Millipore). The inserts were placed in a 24-well plate containing Neurobasal A medium, supplemented with 2% B27 (Invitrogen), 1% N2 (Invitrogen), 2 mM GlutaMAX (Invitrogen), penicillin at a concentration of 100 U/mL, and streptomycin at 100 μg/mL. After 24 h, retinal explants were treated with 0.8 nM of the MerTK inhibitor UNC2025 (Cayman Chemicals, Cat. # 50-296-5010) for 24 h, followed by fixation with 4% PFA and subsequent flatmount immunolabeling for NeuN and Iba-1 markers.

### Primary microglia isolation

Primary microglia were isolated from whole brains of postnatal day 3 (P3) im-HDAC3^−/−^ and HDAC3^fl/fl^ pups using the Neural Tissue Dissociation Kit and CD11b MicroBeads (Miltenyi Biotec) according to the manufacturer’s instructions. Magnetic separation was performed using LS columns and a MidiMACS™ Separator (Miltenyi Biotec). Cells were maintained at 37 °C in high-glucose DMEM supplemented with 10% FBS (Gibco), 10% L929-conditioned medium, and 100 IU/mL penicillin-streptomycin. A 50% media change was performed on day 5 of culture. To induce Cre-mediated recombination and HDAC3 deletion, microglia were treated with 1.5 µM 4-hydroxytamoxifen (4-OHT) on day 3 post-isolation.

### Western blotting

Western blotting was conducted as we previously described [17]. The following antibodies were used: MerTK (R&D Systems, Cat. # AF591), HDAC3 (Proteintech, Cat. # 10255-1-AP), β-actin (Sigma, Cat. #A5441), and ODC (Abcam, Cat. # ab97395). Secondary antibodies (Invitrogen) were prepared in 5% milk in a 1:2000 dilution. Full uncropped blots are provided in Supplementary Fig. S7.

### Statistical analysis

Statistics were performed and graphs prepared using GraphPad Prism 10 software. A *p* value < 0.05 was considered statistically significant. All statistical analyses were performed using the Student’s *t* test (for two-group comparisons) or analysis of variance (ANOVA) with Tukey’s post hoc test (for comparison of multiple groups). Data were presented as mean ± standard deviation (SD). Sample sizes were determined based on previous studies and preliminary experiments to ensure adequate statistical analysis.

### Ethics approval and consent to participate

The animal studies received approval from the Institutional Animal Care and Use Committee (IACUC) of the University of Arkansas for Medical Sciences (UAMS). They were performed in accordance with the Association for Research in Vision and Ophthalmology (ARVO) guide for the care and use of laboratory animals.

## Supplementary information

Supplemental material

## Data Availability

Data are available upon reasonable request.
