# Supplementary material for: Myeloid HDAC3 deletion protects against traumatic optic injury
Source: Cell Death Discov. 2026 Mar 18;12:163. doi: 10.1038/s41420-026-03030-0 (PMC13039952; doi:10.1038/s41420-026-03030-0)
Supplement: Supplementary file 1 — Supplemental material [file 41420_2026_3030_MOESM1_ESM.pdf]

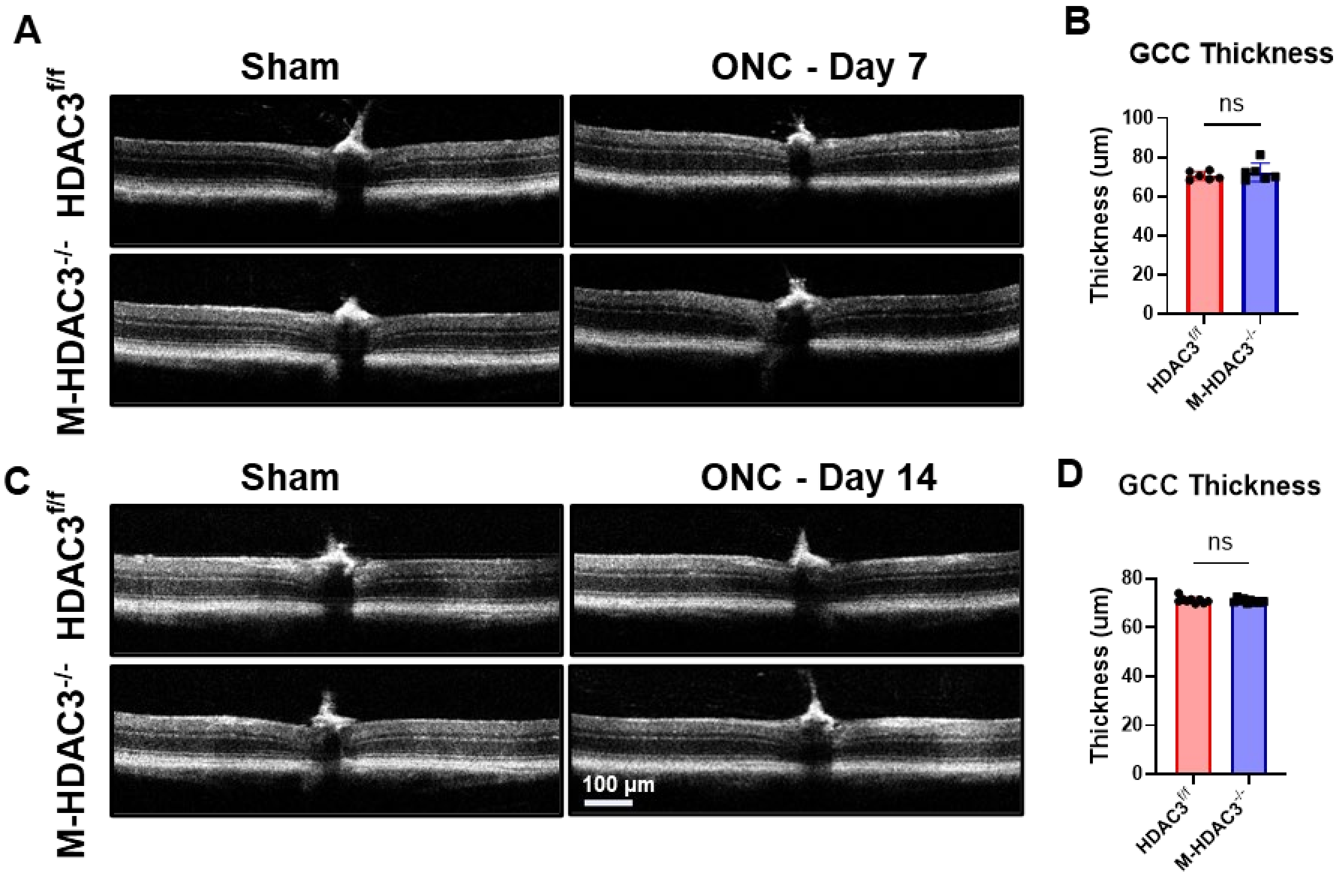

Figure S1. OCT data from sham and injured M-HDAC3$^{-/-}$ and HDAC3$^{f/f}$ mice. Optical coherence tomography (OCT) performed on anesthetized mice at 7 **(A, B)** and 14 days **(C, D)** post-ONC shows no improvement in ganglion cell complex (GCC) layer thickness in M-HDAC3$^{-/-}$ retinas compared to control HDAC3$^{f/f}$. **B**, N = 6; **D**, N = 9-10 per group.

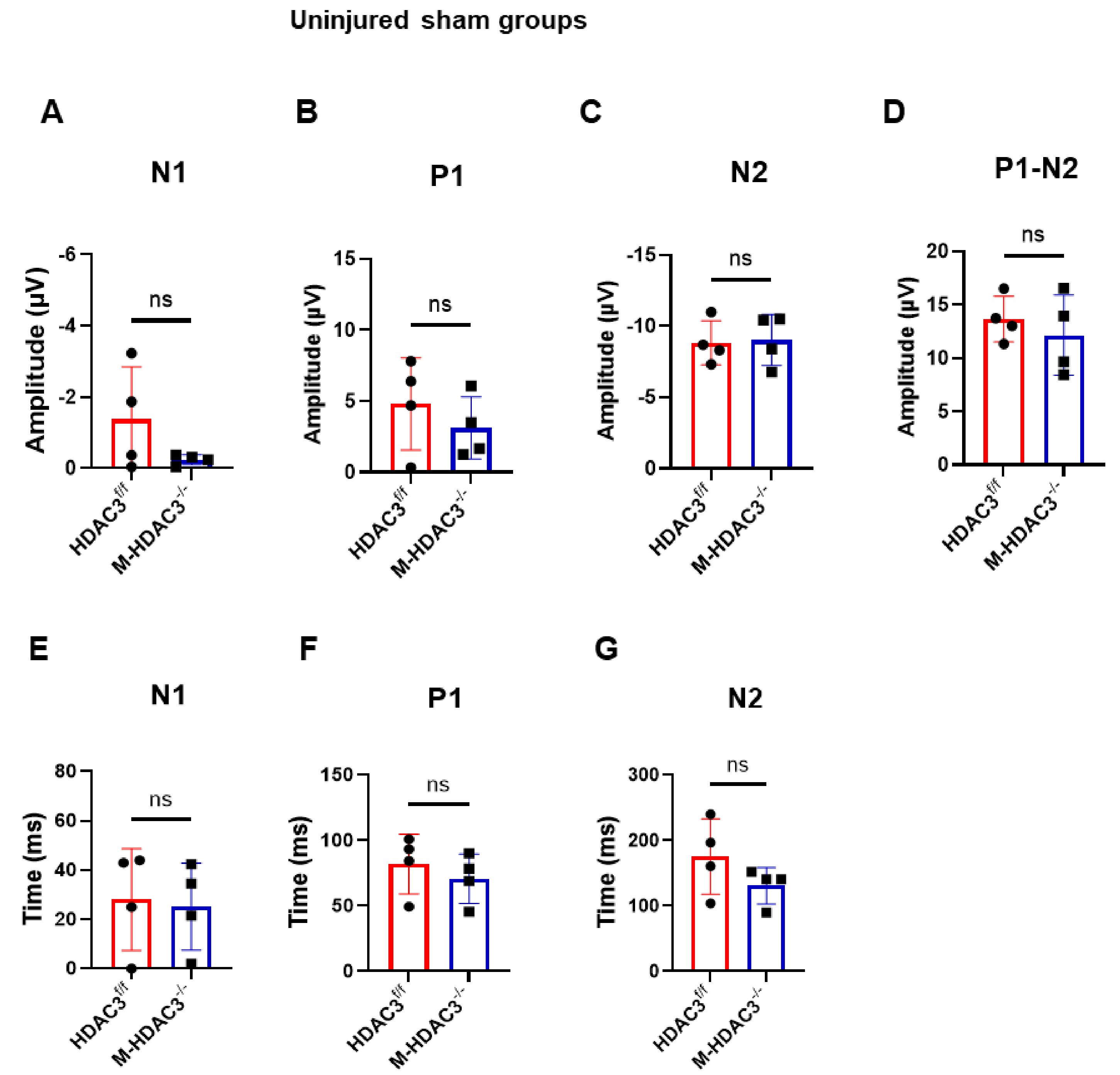

Figure S2. PERG data from M-HDAC3$^{-/-}$ and HDAC3$^{f/f}$ sham mice. Quantification of N1, P1, and N2 waveforms from PERG conducted on HDAC3$^{f/f}$ and M-HDAC3$^{-/-}$ sham mice demonstrates no significant impact of myeloid HDAC3 deletion on the amplitude of N1, P1, and N2 **(A-D)**, nor on their latencies **(E-G)**. N = 4 per group.

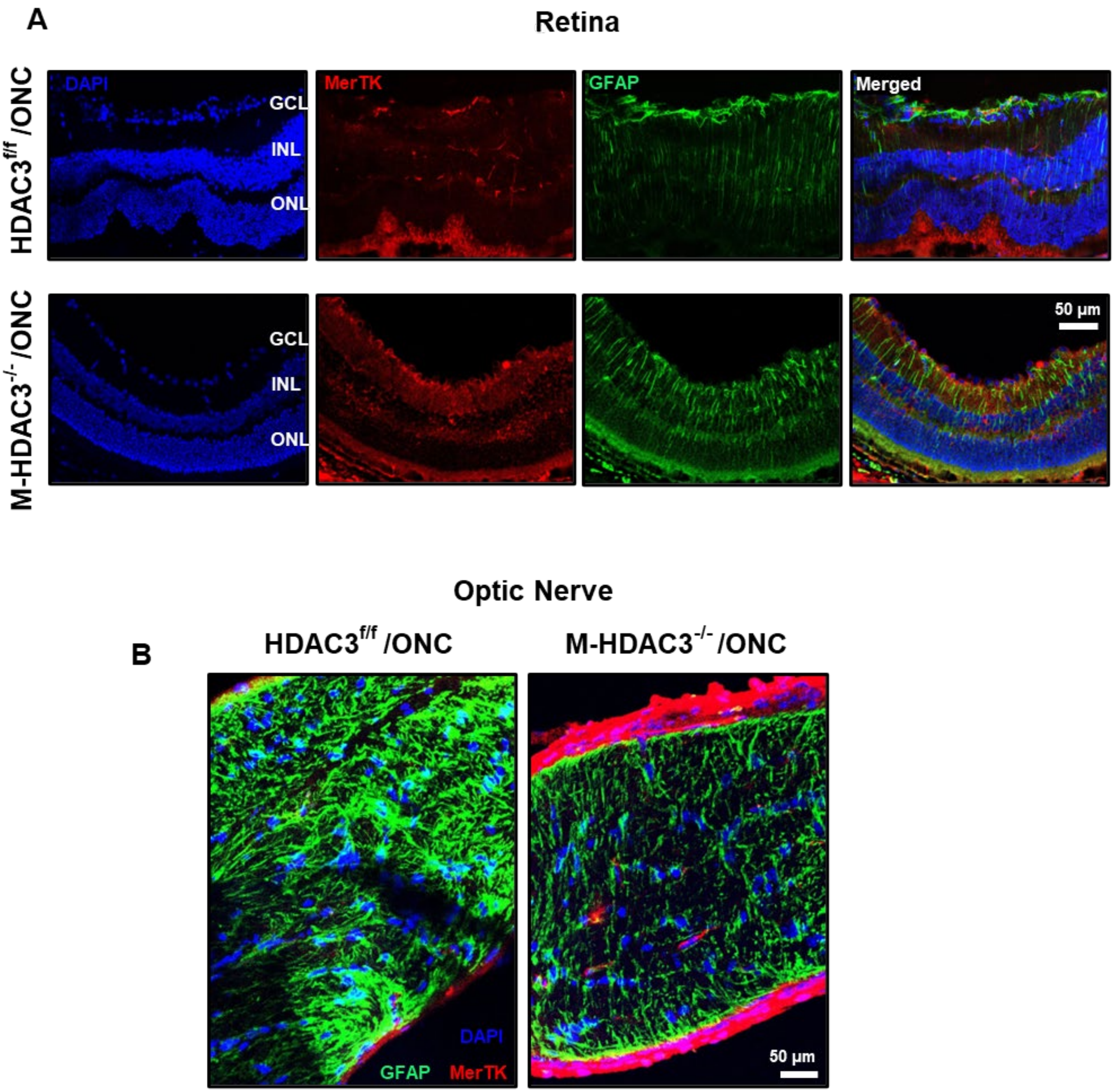

Figure S3. MerTK expression in glial cells following ONC injury. (A) Representative confocal images of glial cells expressing GFAP (green) in the retinas of HDAC3$^{f/f}$ and M-HDAC3$^{-/-}$ mice with minimal colocalization with MerTK (red) at day 5 post-ONC. **(B)** Similarly, in injured optic nerve sections, GFAP and MerTK colocalization were minimal in M-HDAC3$^{-/-}$ and HDAC3$^{f/f}$ control mice at day 5 post-ONC.

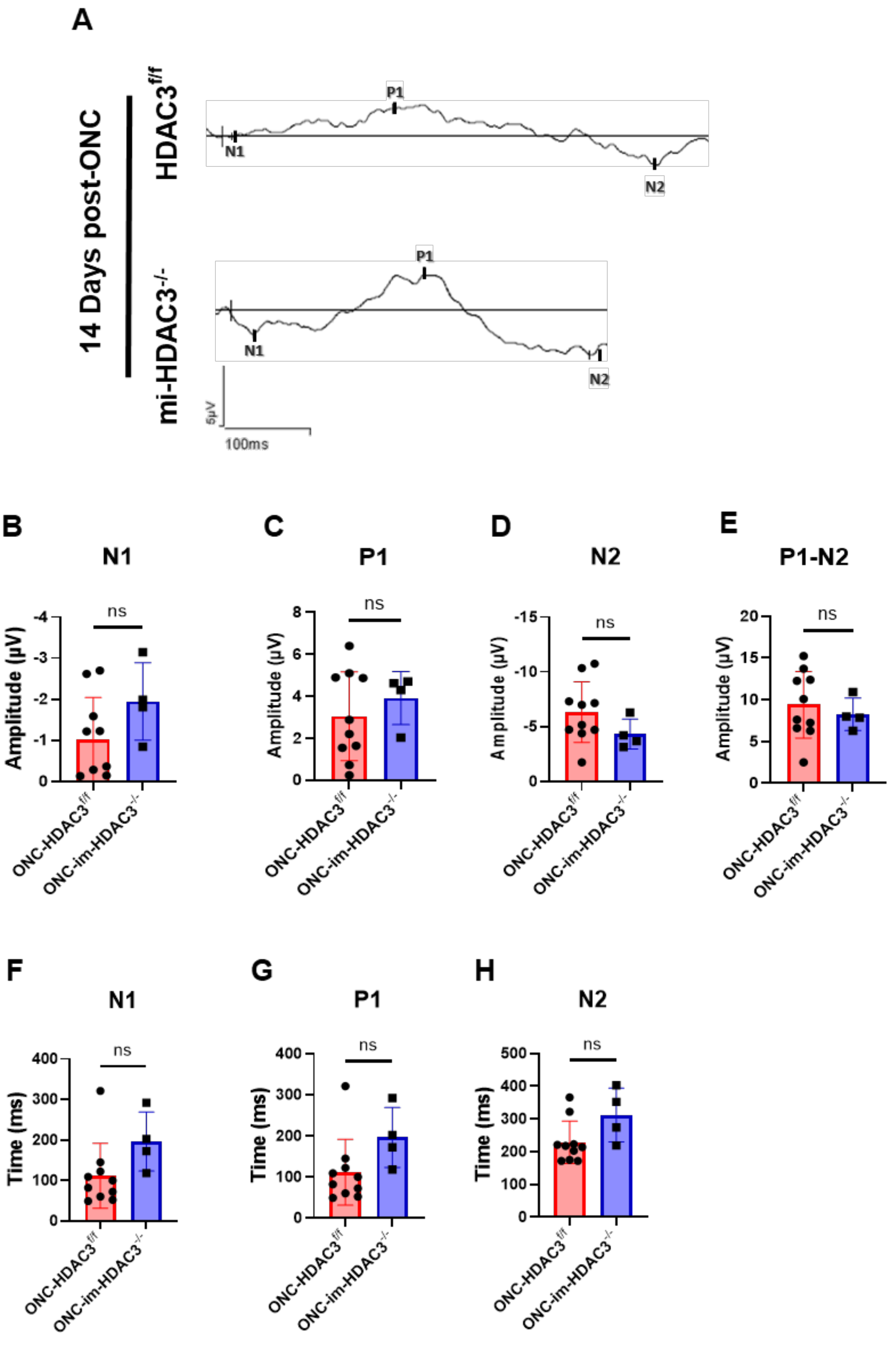

Figure S4. PERG data from sham and injured im-HDAC3$^{-/-}$ and HDAC3$^{f/f}$ mice. (A) Representative of N1, P1, and N2 waveforms in the retinas of microglia-specific HDAC3 KO (im-HDAC3$^{-/-}$) and HDAC3$^{f/f}$ mice 14 days post-ONC. Quantification of N1, P1, and N2 waveforms demonstrates no significant impact of microglia-only HDAC3 deletion on the amplitude of N1, P1, and N2 **(B-E)**, nor their latencies **(F-H)**. HDAC3$^{f/f}$, N = 10; im-HDAC3$^{-/-}$, N = 4.

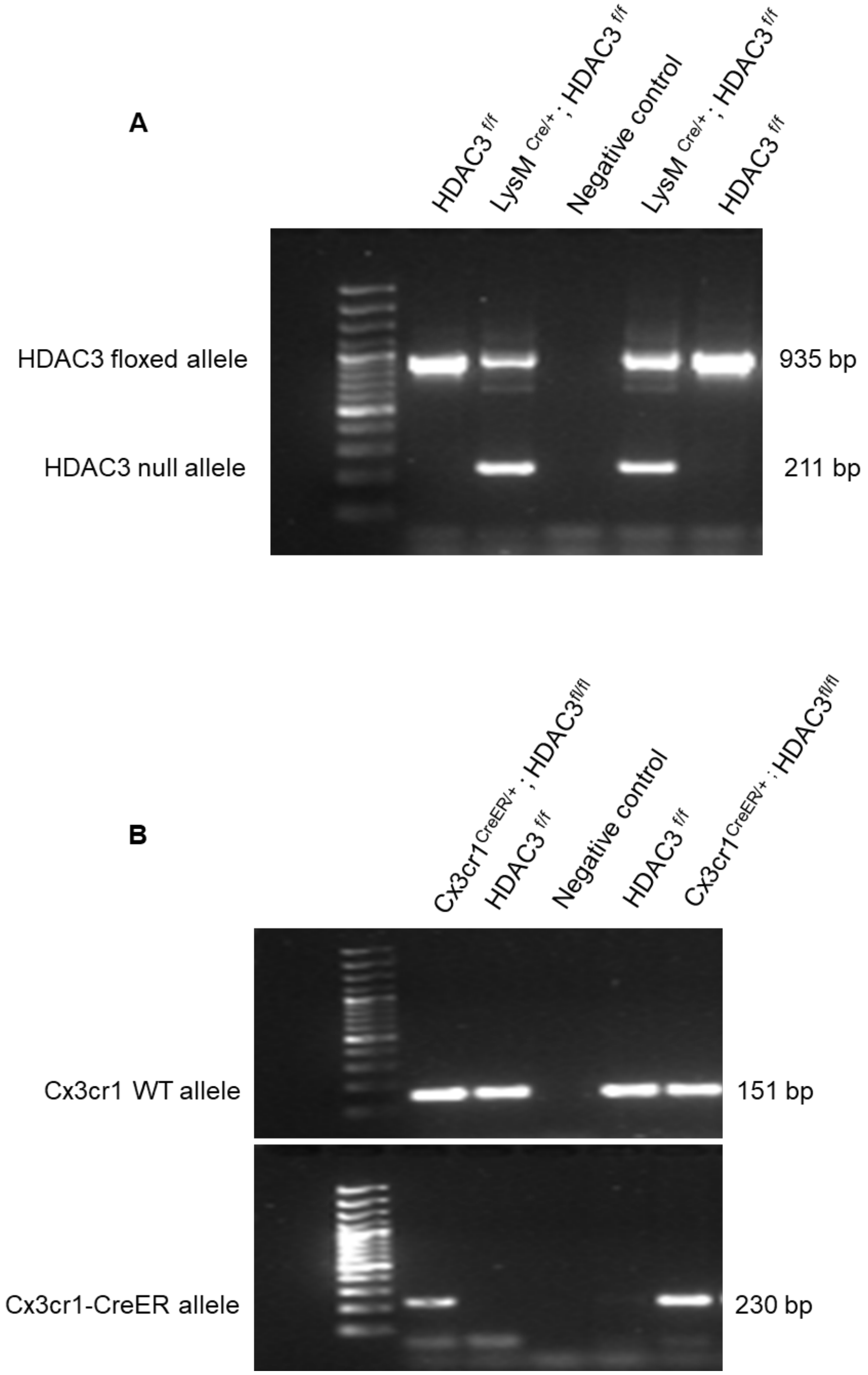

Figure S5. Mouse Genotyping. (A) PCR genotyping of LysM$^{Cre/+}$; HDAC3$^{f/f}$ (M-HDAC3$^{-/-}$) mice shows the Hdac3 floxed allele (935 bp) and the Hdac3 null allele (211 bp). **(B)** PCR genotyping of Cx3cr1$^{CreER/+}$; HDAC3$^{f/f}$ (im-HDAC3$^{-/-}$) mice shows Cx3cr1CreER and WT bands at 230 and 151 bp respectively.

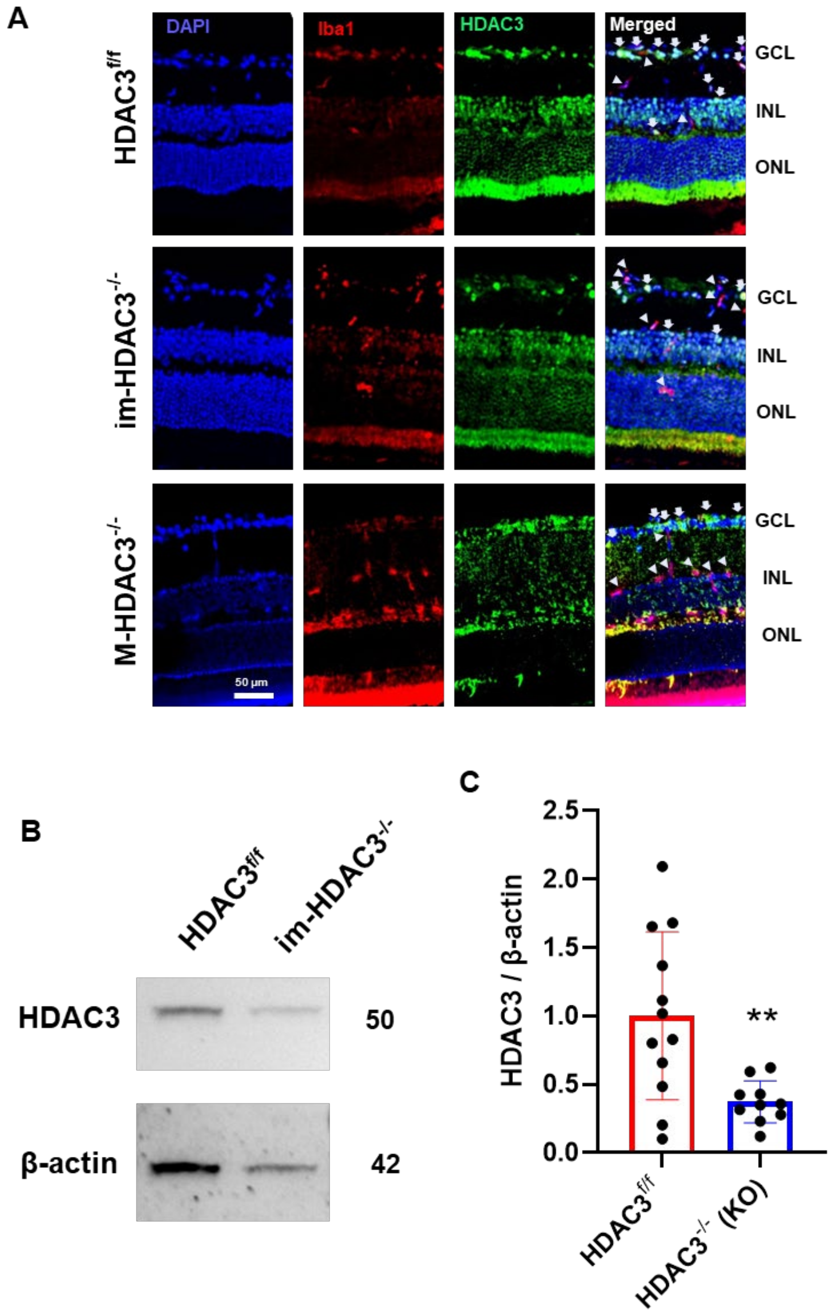

Figure S6. Characterization of M-HDAC3$^{-/-}$ and im-HDAC3$^{-/-}$ mice. (A) Immunolabeling of retina sections from M-HDAC3$^{-/-}$ and im-HDAC3$^{-/-}$ mice for HDAC3 and the microglia/macrophage marker Iba-1 at day 7 after ONC. Arrows show colocalization, whereas arrowheads show Iba-1+ cells lacking HDAC3. M-HDAC3$^{-/-}$ results in HDAC3 deletion in macrophages and 30% of microglia, whereas im-HDAC3$^{-/-}$ results in microglial deletion only. **(B)** Western blotting on primary cortical microglia isolated from Cx3cr1$^{CreER/+}$; HDAC3$^{f/f}$ (im-HDAC3$^{-/-}$) mouse pups and treated with 4-hydroxytamoxifen (4-OHT) to induce HDAC3 deletion. N = 10-12, **$p<0.01$.

Uncropped blots for Figure 6 D in the main manuscript

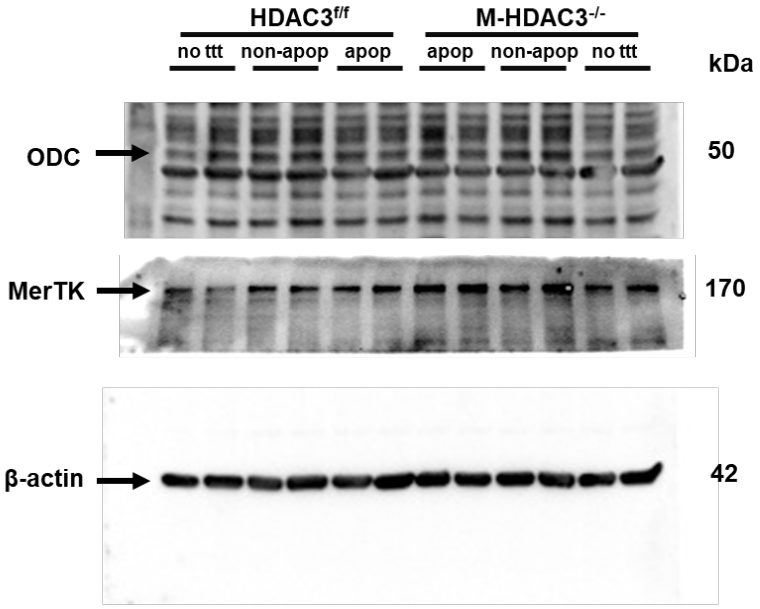

Uncropped blots for Supplementary Figure 2 B

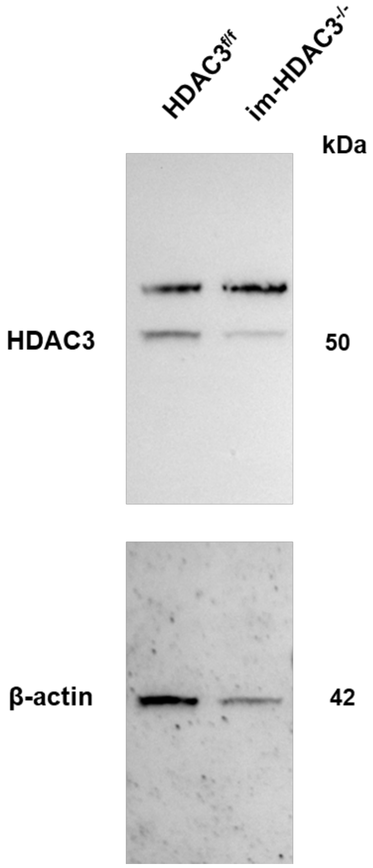

Figure S7. Uncropped blots.
